# SEOM clinical guidelines for the treatment of follicular non-Hodgkin’s lymphoma

**DOI:** 10.1007/s12094-015-1437-1

**Published:** 2015-11-19

**Authors:** M. Provencio Pulla, J. Alfaro Lizaso, L. de la Cruz Merino, J. Gumá i Padró, C. Quero Blanco, J. Gómez Codina, M. Llanos Muñoz, N. Martinez Banaclocha, D. Rodriguez Abreu, A. Rueda Domínguez

**Affiliations:** Servicio de Oncología Médica, Hospital Universitario Puerta de Hierro Majadahonda, Madrid, Spain; Servicio de Oncología Médica, Instituto Oncológico de Guipúzcoa, San Sebastián, Spain; Servicio de Oncología Médica, Complejo Hospitalario Regional Virgen Macarena, Sevilla, Spain; Servicio de Oncología Médica, Hospital Universitari de Sant Joan de Reus, Reus, Spain; Servicio de Oncología Médica, Complejo Hospitalario Regional y Virgen de la Victoria, Málaga, Spain; Servicio de Oncología Médica, Hospital Universitari i Politècnic la Fe, Valencia, Spain; Servicio de Oncología Médica, Hospital Universitario de Canarias (H.U.C), San Cristóbal de La Laguna, Tenerife Spain; Servicio de Oncología Médica, Hospital General Universitario de Elche y Vega Baja, Elche, Spain; Servicio de Oncología Médica, Hospital Universitario Insular de Gran Canaria, Las Palmas de Gran Canarias, Spain; Área de Oncología y Hematología, Hospital Costa del Sol, Marbella, Spain

**Keywords:** Oncohematology malignancies, Follicular non-Hodgkin’s lymphoma, Non-Hodgkin lymphoma therapy

## Abstract

Follicular non-Hodgkin’s lymphoma (FL) is a nodal B lymphoid malignancy that originates from the germinal center of a lymph node. FL is the second most frequent lymphoma subtype. The course of the disease is usually characterised by a typically indolent clinical course, with a median survival rate of 8–10 years, although most patients relapse after treatment. Diagnosis should always be based on a surgical specimen like an excisional node lymph biopsy. The first-line treatment of FL will depend of extension disease, tumour burden, patient symptoms, performance status and also patient decision. The addition of rituximab to conventional chemotherapy has improved ORR, PFS and OS. As first line in patients that need treatment, a combination of chemotherapy with rituximab induction followed by 2 years of rituximab maintenance is the best option. High-dose chemotherapy with autologous stem-cell transplantation in first line has not shown improvement and is not recommended as first-line therapy. Before any treatment decision in relapsed patients, a repeat biopsy is mandatory to rule out a transformation into large cell aggressive lymphoma. Standard treatment is controversial, depends on the efficacy of prior treatment, duration of the time-to-relapse, patient’s age and histological findings at relapse.

## Methodology

To identify the main topics published in medical literature, a search in "PubMed" and "is knowledge"(that includes both full papers and abstracts) has been performed. Key words used were "Non- Hodgkin Lymphoma", "Follicular Lymphoma staging" "Follicular Lymphoma treatment", and "Follicular Lymphoma new therapies".

Main recent reviews on the topics: ESMO clinical guides, NCCN guides, Annual Clinical Updates in Hematology Malignances of the American Journal of Hematology, have been consulted.

## Introduction

Follicular non-Hodgkin’s lymphoma (FL) is a nodal B lymphoid malignancy that originates from the germinal centre of a lymph node [1]. FL is the second most frequent lymphoma subtype, and in recent decades, the incidence has risen to 5–7 cases per 100,000 people and represents approximately 20 % of all lymphomas.

Age-adjusted rate according to European Registry HAEMACARE is 2.18 (IC 95 % 2.12–2.24) per 100.000 people [2]. In a Spanish study conducted between 1999 and 2009, 3651 lymphoid malignancies were registered, 18 % of which were FL, and were the second most common subtype [3].

Most patients with FL suffer from the disease in their sixties or seventies, and it rarely occurs in younger people. The course of the disease is usually characterised by a typically indolent clinical course, with a median survival rate of 8–10 years, although most patients relapse after treatment [4].

A spontaneous regression of the disease has been reported in nearly 25 % of patients. In many cases, the terminal phase of the disease is associated with transformation to aggressive lymphoma, with an incidence ranging from 16 to 60 %.

## Diagnosis

Diagnosis should always be based on a surgical specimen like an excisional node lymph biopsy. Core biopsy should only be performed in patients without accessible lymph nodes (e.g. retroperitoneal node). Fine needle aspirations are inappropriate for a reliable diagnosis.

FL is characterised by a follicular growth pattern, usually composed of a mixture of centrocytes and centroblasts. Grading is performed according to the number of centroblasts observed in a high-power field, and includes grades 1–2 (≤15 blasts), 3 (>15 blasts), 3a (centrocytes still present) and 3b with sheets of centroblasts. FL grade 3b is considered to be an aggressive lymphoma.

FL has a characteristic immunophenotype, which includes CD19, CD20, CD22, CD79a+, surface immunoglobulins (sIg: IgM, IgD and IgG), Bcl-2+, CD10±, CD5− and CD43−. The chromosome translocation t(14:18) (q32;q21), which juxtaposes the bcl-2 gene with the immunoglobin heavy-chain locus, deregulates the expression of the BCL-2 gene in 90 % of FL grade 1 and 2 [5].

In grade 3 FL lacking t (14; 18) it is recommended to perform the study for BCL-6, as it can also be useful in pediatric FL. Gene expression profiles are being studied both in lymphoma cells and in their microenvironment, but they are currently not in use in daily clinical practice [6].

There are three rare clinicopathological variants:

- *Bowel primary FL* more common in the second portion of the duodenum, in the form of multiple small asymptomatic polyps. Most patients have a localised disease (stage IE or IIE).
- *Cutaneous primary FL* solitary lesion or few localised lesions (only 15 % presents generalised lesions) in head and trunk (typically, in the back).
- *Pediatric FL* nodal and extranodal involvement (Waldeyer ring and testicles). It owns unique clinicopathological features: very large follicles, blastoid cytologic features, high proliferation rate and lack of expression of BCL-2 and t(14; 18) (q32; q21). It comes in early stages and is generally associated with a good prognosis.

## Staging

The diagnostic work-up of FL is similar to other lymphomas. Initial work-up should include clinical history and physical examinations, paying special attention to the lymph nodes, liver and spleen; a complete blood count; routine blood chemistry, including liver and renal function, lactate dehydrogenase (LDH) levels, uric acid levels, immunoglobulin levels and β2 microglobulin levels; as well as screening tests for HIV, hepatitis B and hepatitis C. A computed tomography (CT) of neck, thorax, abdomen and pelvis and bone marrow biopsy have to be performed.

The recent Consensus of the International Conference Lymphoma Working Group recommends performing PET-TC [7]. In case of histology transformation suspicion, PET can identify the optimal site for biopsy. It is also useful in the early stages that will be treated with radiation therapy to confirm localised disease [IV, C].

FL staging is typically given according to the Ann Arbor system [8]. (Table 1). Lugano Classification no longer recommends the addition of B symptoms or the use of X for bulky disease in FL [9].

**Table 1:** Cotswolds/Ann Arbor staging system

Stage I	Single lymph node group or extranodal (IE)
Stage II	Multiple lymph node groups on the same side of the diaphragm or extranodal and one or more lymph node (IIE) on the same side of the diaphragm
Stage III	Multiple lymph node groups on both side of the diaphragm
Stage IV	Presence of diffuse or disseminated involvement of one or more extralymphatic organs

Most patients present advanced stage lymphoma, directing the need for more information for prognostic purposes. The Follicular Lymphoma-specific International Prognostic Index (FLIPI) has established 5 risk factors: more than four of these factors involve node sites, elevated LDH levels, age >60 years, advanced III–IV stage and haemoglobin levels <12 g/dl. FLIPI stratifies patients into three different risk categories: low (0–1), intermediate (2) and high (3–5) risk for overall survival, with different rates of survival [10] [I, A] (Table 2).

**Table 2:** Follicular lymphoma international prognostic index (FLIPI)

FLIPI score	No. of risk factors	Patients (%)	5 years survival (%)	10 years survival (%)
Good	0–1	36	90.6	70.7
Intermediate	2	37	77.6	50.9
High	3–5	27	52.5	35.5

FLIPI-2 [11] has been developed in a prospective study of patients treated with rituximab. Factors that include FLIPI-2 are the following: age >60 years, bone marrow infiltration, haemoglobin <12 g/dl, high β2microglobulin and lymph node diameter >6 cm.

## Treatment

### First-line treatment

**Fig. 1:**
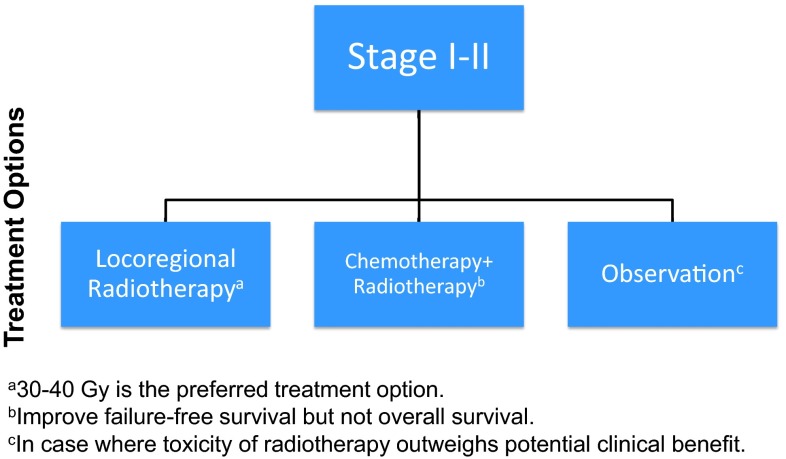
Treatment algorithm of follicular lymphomas (Grade 1–2) localised disease

The first-line treatment of FL will depend on the extension of the disease, tumour burden, patient symptoms, performance status (PS) and also the patient's decision. (Fig. 1).

### Stage I–II disease

Only 15–25 % of patients are diagnosed with non-bulky Ann Arbor stage I/II. In asymptomatic patients, initial observation is a valid option [IV, B] [12, 13]. In symptomatic patients, Involved Field Radiation (IFR) (24–36 Gy) is the most recommended treatment worldwide, achieving complete responses (CR) up to 97 % of cases and long-term disease control with most recurrences outside radiation field [II, B] [14, 15]. However, there are no randomised studies against other strategies and patients involved in the studies with radiation therapy are heterogeneous with different doses and schedules used over the time. The addition of chemotherapy has not demonstrated any further benefits and anti-CD20 therapy has not been adequately studied in limited-stage FL [IV, C] [16]. Observation or Rituximab (R) monotherapy may be an option to avoid radiotherapy side effects with no deleterious impact in overall survival (OS) [IV, B] [17]. In cases of high tumour burden, patients can be treated with chemoimmunotherapy like in the advanced setting before radiation therapy [IV, B] [18].

### Stage III–IV disease

**Fig. 2:**
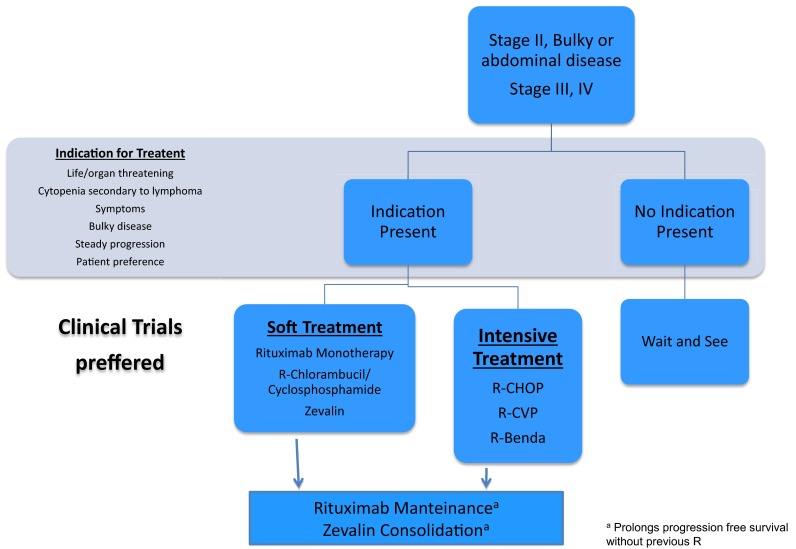
Treatment algorithm for advance disease

To date, advanced stage FL was considered incurable with conventional treatment strategies. But recently, the plateau observed in the survival curves at 10 years in different series indicates the existence of long-term survivors in the R era. Therefore, the goal of treatment is to achieve the best response and prolong progression-free survival (PFS) and, if possible, prolong OS (Fig. 2).

The decision to treat and the selection of treatment should be individualised based on the patient symptoms, tumour burden, hematopoietic impairment, histological transformation and comorbid conditions. In general, GELF (Groupe pour l’Etude de Lymphome Folliculaire) criteria are used to initiate treatment [19] (Table 3).

**Table 3:** GELF Criteria

∙ Any nodal or extranodal tumour mass (except spleen) with a diameter >7 cm
∙ Involvement of more than three nodal sites with a diameter >3 cm
∙ Presence of systemic symptoms
∙ Substantial splenic enlargement (symptomatic or >16 cm on computed tomography)
∙ Pleural or peritoneal effusions
∙ Risk of local compression (epidural, intestinal, ureteral and orbital among others)
∙ Circulating lymphoma cells (>5 × 109/l malignant cells)
∙ Marrow compromise (haemoglobin <10 g/dl, granulocytes count <1.5 × 109/l or platelet count <100 × 109/l
∙ ECOG PS ≥2
∙ Elevated serum LDH or B-2 microglobulin

Both before the era of R and at present, in asymptomatic patients with low tumour burden, the strategy of “wait and see” has had no negative impact on patient survival as demonstrated in different randomised trials [16]. So, taking into account that median age of advanced FL patients is between 60 and 65 years and spontaneous regressions are described in about 10–20 % of cases, asymptomatic patients can be managed with a wait and see strategy [I, A].

Patients with indication for treatment should be treated with systemic therapy and, if not contraindicated, associated with R. Inclusion in clinical trials with new agents should alwaysobservation is a valid opti be considered as the first line of treatment.

The addition of R to conventional chemotherapy has improved ORR, PFS and OS in different trials and one meta-analysis, including first line and salvage therapy settings [20] [I, A]. R-CHOP schedule is the most widely used worldwide but other schemes also can be used according to patient characteristics without significant differences in overall survival [21, 22] [I, B]. The use of fludarabine has been declining because of drug toxicity profile that may hinder the further management of the patient. The combination of R-Bendamustine as induction therapy has demonstrated increased PFS and OS compared with R-CHOP in first-line treatment of advanced LF in a phase III study. However, this study was performed without maintenance R so it is unclear whether induction with R- Bendamustine is comparable to treatment with R-CHOP induction plus 2 years of maintenance with R [23] [I, B] (Table 4).

**Table 4:** First line: Different schemes of chemoimmunotherapy

Combination	ORR (%)	PFS 3y (%)	Neutropenia 3, 4 (%)
R-CVP	88, 91	46, ND	28, 56
R-CHOP	93, 91	62, ND	50, 87
R-FM	91	59, ND	64
R-B	97	ND	39

*R-CVP* rituximab, cyclophosphamide, vincristine, prednisolone; *R-CHOP* rituximab, doxorubicin, cyclophosphamide, vincristine, prednisolone; *R-FM* rituximab, fludarabine, mitoxantrone; *R-B* rituximab, bendamustine; *ND* no data; *ORR* overall response rate; *PFS* progression-free survival

Regarding maintenance therapy, the results from the Primary Rituximab and Maintenance (PRIMA) phase III trial have demonstrated that maintenance therapy results in longer PFS, although there is no impact in overall survival (OS) [24] [I, B]. A meta-analysis suggested the use of interferon maintenance therapy as part of first-line treatment [II, B]; however, due to poor toxicity profile front of R has made the latter preferred in this setting [III, B].

Radioimmunotherapy consolidation prolongs PFS after chemotherapy, although its benefit following R combinations has not been established as a first-line treatment [25]. It can be an option for patients who are unable to tolerate standard chemotherapy (elderly or unfit patients) or in high-risk patients achieving a PR or CR after induction therapy [II, B].

In conclusion, as first line in patients that need treatment, a combination of chemotherapy with rituximab induction followed by 2 years of rituximab maintenance is the best option [I, B].

For low-risk or unfit patients, the use of single agents (chlorambucil and cyclophosphamide), R monotherapy, radioimmunotherapy, or a combination of R-chlorambucil and R-cyclophosphamide is a good choice to consider [III, B].

## Radiation therapy is only used for palliation in locally symptomatic disease [II, B]

High-dose chemotherapy with autologous stem-cell transplantation in first line has not shown improvement and is not recommended as first-line therapy, this strategy should be used only in clinical trials [26] [I, D].

### Second-line treatment

**Fig. 3:**
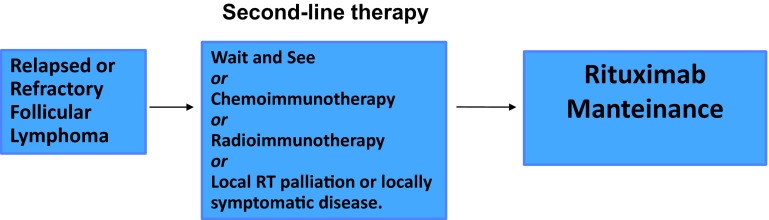
Treatment algorithm for relapsed or refractory follicular lymphoma

Before any treatment decision in relapsed patients, a repeat biopsy is mandatory to rule out a transformation into large cell aggressive lymphoma (Fig. 3).

Clinical trial participation is always recommended in relapsed or refractory follicular lymphoma. Standard treatment is controversial, and depends on the efficacy of prior treatment, duration of the time-to-relapse, patient’s age and histological findings at relapse.

Retreating with the same chemotherapy that was effective is reasonable especially after a long remission. Nevertheless, a non-cross-resistant scheme should be preferred (bendamustine after CHOP) [I,B]. The addition of rituximab should be considered if previously it was able to achieve a durable remission and then to continue with rituximab maintenance every 3 months which has demonstrated to significantly improve OS, compared with observation or treatment with the drug at the time of disease progression, according to a new meta-analysis [I, A].

Radioimmunotherapy has been demonstrated to be an effective second-line treatment even in patients who have FL recurrence after rituximab exposure [27] [I, B].

High-dose chemoradiotherapy with autologous hematopoietic cell transplantation is effective in relapse FL but its role has to be redefined in the rituximab era. Allogeneic stem-cell transplantation can provide durable long-term molecular remission but with high treatment-related mortality, reserving this modality for young and very motivated patients, with suitable donors [28].

Different promising new agents are currently being explored for the treatment of patients with relapsed or refractory FL, new antibodies (targeting CD20, CD 22 and CD23, such as Obinutuzumab, epratuzumab and lumiliximab); new drugs targeting oncogenic pathways (PI3K, such as idelalisib or Bruton´s tyrosine kinase inhibitors such as ibrutinib); immunotherapy (anti-PD1 as Pidilizumab).
